# Single compound data supplementation to enhance transferability of fermentation specific Raman spectroscopy models

**DOI:** 10.1007/s00216-025-05768-5

**Published:** 2025-02-06

**Authors:** Maarten Klaverdijk, Marcel Ottens, Marieke E. Klijn

**Affiliations:** Department of Biotechnology, Delft University of Technology, Van Der Maasweg 9, Delft, 2629 HZ The Netherlands

**Keywords:** Raman spectroscopy, Chemometrics, Partial least squares (PLS), *Saccharomyces cerevisiae*, Real-time monitoring

## Abstract

Raman spectroscopy is a valuable analytical tool for real-time analyte quantification in fermentation processes. Quantification is performed with chemometric models that translate Raman spectra into concentration values, which are typically calibrated with process data from multiple comparable fermentations. However, process-specific models underperform for minor process variation(s) or different operation modes due to the integration of cross-correlations, resulting in low target analyte specificity. Thus, model transferability is poor and labor-intensive (re-)calibration of models is required for related processes. In this work, partial least-squares models for glucose, ethanol, and biomass were calibrated with *Saccharomyces cerevisiae* batch fermentation data and subsequently transferred to a fed-batch operation. To enhance model transferability without additional process runs, single compound data supplementation was performed. The supplemented models increased overall target analyte specificity and demonstrated sufficient prediction accuracy for the fed-batch process (root-mean-square errors of prediction (RMSEP) of 3.06 mM, 8.65 mM, and 0.99 g/L for glucose, ethanol, and biomass), while maintaining high prediction accuracy for the batch process (RMSEP of 1.71 mM, 4.20 mM, and 0.17 g/L for glucose, ethanol, and biomass). This work showcases that process data in combination with single compound spectra is a fast and efficient strategy to apply Raman spectroscopy for real-time process monitoring across related processes.

**Supplementary Information:**

The online version contains supplementary material available at 10.1007/s00216-025-05768-5.

## Introduction

Bioprocess development, optimization, and control are dependent on various information sources during fermentation, ranging from pH and temperature to cell viability and product concentration. State-of-the-art bioreactor processes already include automated measurements and coupled control loops for decades, but these are often limited to parameters such as temperature, pH, dissolved oxygen (DO), and off-gas analysis [1]. For information on metabolite concentration, product quality, or the biomass, labor-intensive manual sampling and analysis is performed by trained process technicians. Accurate monitoring of all process parameters during fermentation is considered essential to ensure fast bioprocess development and stable manufacturing. Although off-line analytics are the golden standard for investigating complex parameters, manual sampling required for these measurements causes a limited and delayed perspective of the process. This leads to retrospective decision-making and the inability to pro-actively make process adjustments to ensure a successful run.

The bioprocessing industry is actively investigating process analytical technologies (PAT) that provide detailed information on a wide(r) range of critical process and product parameters, allowing the transition to data-driven bioprocess development and automated decision-making [2]. Robust real-time quantification of metabolite, product, and biomass concentrations can lead to more efficient bioprocess development and serve as response values required for automated bioprocess control. There are many different analytical techniques available for abovementioned parameters, such as enzyme-based biosensors, impedance-based probes, capacitance probes, and optical spectroscopy tools [3–5]. Among the available technologies, in-line optical spectroscopy tools that capture molecular vibrations have the advantage of being non-invasive, non-destructive and provide continuous measurements [6]. Raman spectroscopy is a suitable choice for aqueous systems as seen in bioreactors, due to the low signal interference from water. Moreover, Raman spectroscopy captures spectral contributions of multiple analytes present in a bioreactor in a single spectrum, thereby facilitating efficient multiplexed quantification. The obtained Raman spectra are correlated to reference measurements from off-line analytics to translate the complex information into quantitative metrics required for bioprocess development and control. This is achieved using chemometric methods, which focus on studying the relationship between chemical measurements and the properties of interest during a process [7]. Chemometric methods in combination with Raman spectroscopic data have been employed for in-line monitoring and quantification of substrates, products, waste-products, and cell density in a wide range of processes, such as ethanol production by yeast and antibody production with CHO cell lines [8–10].

Although different chemometric methods are reported on, partial least square (PLS) regression is the most used technique for calibrating Raman spectroscopy quantification models in bioreactor applications [10–12]. Raman spectra contain many spectral variables (wavenumbers), while there is often only a single response parameter (reference measurement) per analyte [12, 13]. PLS models are considered appropriate for such datasets, described as systems containing high numbers of collinear predictor variables and limited response values [14]. The method is widely available in statistical software packages and allows the development of quantification models without needing extensive process knowledge. By using Raman spectra obtained during fermentation and orthogonal off-line measurements on an analyte of interest as input data, the PLS model is calibrated by assigning weights to the relevant spectral variables for its quantification. The calibrated model can be interpreted through the regression coefficients and loadings of the generated latent variables. However, an appropriate calibration dataset should capture data over the full process range, contain process and biological variability, and preferably contain an even sample distribution to prevent possible accuracy biases. As the biological processes which we want to monitor are subject to inherent cross-correlations between the changes of substrate, product, and biomass concentrations, cross-correlations are directly incorporated into the calibration datasets [15]. This is a challenge for calibrating robust PLS models, as it is an implicit modeling technique that maximizes covariance between the X (Raman spectra) and Y (reference method) data and has no knowledge of the system. The strong cross-correlations between the response values can cause a PLS model to become non-specific, meaning that information from one compound (e.g., substrate) is used to quantify a cross-correlated compound (e.g., product or biomass). This subsequently leads to non-specificity of models towards the analyte of interest. This lack of analyte specificity may be less important in cases where the relationship between the cross-correlated parameters does not change (e.g., monitoring identical processes) or when the goal is to monitor solely process evolution. However, as these models are tailored to a specific process, the predictive capability is compromised when these models are applied to processes where the relation between the parameters changes [16]. This can limit the potential of continuous monitoring and control in a dynamic research and development environment, where aspects such as feeding strategies, mode of operation, or inoculation density can still be subject to change. As a consequence, upon the need of monitoring related processes, the extensive data collection and model calibration procedure has to be repeated. Whereas the collection of in-line Raman spectra has been automated with recent technological advancements, the collection of high-quality measurements required as orthogonal reference data is material- and labor-intensive. Furthermore, high-quality process data is not directly available when changing process parameters, meaning that Raman spectroscopy monitoring is not available until the data of several new process runs is collected. Next to that, the effects of a changed process parameter on the concentration ranges of compounds of interest are not always known beforehand. This limits the implementation speed and impact of real-time monitoring with Raman spectroscopy in dynamic environments, where live process data could be essential to early process understanding. Therefore, it is desired to efficiently develop robust models, to ensure model predictive performance remains unaffected by (minor) variations in manufacturing and environmental conditions.

To prevent the adverse effects of highly cross-correlated process data on the performance of PLS models across related processes, calibration datasets should be adjusted accordingly. Alternative approaches for data collection to supplement regular process data include target compound spiking (i.e., analyte spiking), generating synthetic spectra, and combining these approaches with design of experiments (DoE). For the first approach, spiking, the level of cross-correlation in a calibration dataset is evaluated and subsequently disrupted by supplementing additional samples. For example, a cross-correlation between substrate consumption and product formation can be disrupted by spiking the substrate in the bioreactor to generate conditions that break the cross-correlation observed in the original process. However, spiking compounds during an ongoing at-scale bioprocess is not efficient, as it may impair the continuation of the process. Therefore, measurements of spiked samples are often acquired in smaller scale reactors or shake flasks [15, 17]. The second approach involves the generation of synthetic samples outside of the bioreactor process to mimic process conditions. Samples can either be completely synthetic by externally preparing single compound mixtures of target compounds in cultivation media, or bioreactor samples can be utilized by altering the compound concentrations and performing measurements in miniaturized acquisition setups [18]. DoE techniques are often employed to design samples with uncorrelated compounds [19]. The combination of bioreactor and synthetic spectral data has proven to increase calibration model performance for several processes [15, 19]. However, depending on the complexity of the process and the number of compounds to be considered, these approaches can still lead to material- and labor-intensive calibration dataset preparation. As PLS models are typically calibrated to quantify only one process compound, it is essential that the model identifies and correlates spectral features specific to that single compound. Considering this concept, it may not be necessary to add data containing variation on other compounds, as these are already accounted for by process data in the base dataset. By supplementing the dataset with spectra of the target compound at varying concentrations, spectral features associated with the compound of interest are emphasized, which reduces cross-correlations within the dataset. Through this supplementation approach, models may be improved and extended beyond the calibration ranges of the original dataset based on process data.

This work investigates the applicability of single compound spectra supplementation as an efficient and simple alternative for calibration dataset adjustment to improve PLS model performance across related processes. The yeast *Saccharomyces cerevisiae* was used as a model system to generate batch process data for the calibration of base models for the quantification of glucose, ethanol, and biomass. These base models are subsequently transferred to a fed-batch cultivation where the models have to extrapolate and deal with new process conditions. We demonstrate how standard quantitative model validation is not always sufficient to assess model quality and could lead to poor performance when process parameters change. After a qualitative assessment of the models, the calibration datasets were supplemented with single compound spectra to improve model transferability by increasing the calibration range and target specificity. This work shows the impact of qualitative model assessment and how to efficiently adjust calibration datasets to improve model transferability. This concept of a base model in combination with data supplementation can be used to extend the applicability of Raman spectroscopy models across related processes, without the need for collecting new process data. Methods like these could allow more efficient and flexible model transfer within dynamic process development environments.

## Materials and methods

### Fermentation

The yeast strain *Saccharomyces cerevisiae* CEN.PK113-7D was used for all experiments [20]. All cultures were grown on synthetic medium containing 5 g/L (NH4)2SO4, 3 g/L KH2PO4, and 0.5 g/L MgSO4.7H2O adjusted to pH 6.0 with 2 M KOH [21]. The pH was measured with an offline pH probe (Consort, Turnhout, Belgium). Glucose was used as carbon source, and an initial concentration of 20 g/L was reached by the addition of sterilized 50% glucose (J.T. Baker, Philipsburg, NJ) solution (in-house). Vitamin solution was sterilized through 0.2-μm syringe filters (Whatman, Maidstone, UK) and added after sterilization of the medium. Bioreactor medium was supplemented with 0.2 g/L sterile Antifoam-C (BASF, Ludwigshafen, Germany) after autoclaving.

Pre-cultures were grown aerobically in 500-mL shake flasks with a 100-mL working volume and were incubated at 30 °C and 150 rpm in an orbital shaker (Sartorius, Göttingen, Germany). Batch cultures were grown in 2-L stirred tank reactors (Applikon, Delft, the Netherlands) using a working volume of 1 L. The cultures were aerated with 0.5 L/min air while stirred at 800 rpm and maintained at a temperature of 30 °C. A pH of 6.0 was maintained by the automatic addition of 2 M KOH. Batch cultures were inoculated at an initial OD660 of 0.3. The fed-batch culture started out as a batch process described above, but was spiked with sterile 50% glucose solution when substrate depletion was detected through a decrease in CO_2_ production. CO_2_ production was measured with off-gas analysis with a ServoPRO 4900 (Servomex, Crowborough, UK). The fed-batch culture was fed with 50% glucose solution three times, thereby extending the process duration and increasing the final concentrations of ethanol and biomass. An overview of glucose, ethanol, and biomass concentrations during the performed batch and fed-batch fermentations is provided in Supplementary Figure S5.1.1.

### Single compound solutions

Single compound spectra were acquired in the same 2-L bioreactor as described in the “Fermentation” section to maintain similar acquisition conditions, such as aeration, stirring, and temperature. For each analyte, the bioreactor was filled with 1 L of synthetic media (as described in the “Fermentation” section) with a constant temperature of 30 °C, stirring rate of 800 rpm, and aeration with 0.5 L/min of air. Glucose and ethanol concentration ranges were generated by the stepwise addition of 10 mL prepared glucose or ethanol solution. After acquiring Raman spectra at a concentration, a 10 mL sample was taken for reference measurements by HPLC, and the subsequent concentration was achieved by adding the next 10 mL prepared solution. The 10 mL additions were prepared beforehand according to predefined concentration distributions. The glucose concentrations were increased in an exponentially increasing manner to generate more low concentration conditions. The ethanol concentration was increased with steps of 20 mM. To generate biomass single compound spectra, a batch process was operated until glucose depletion occurred, after which the full bioreactor volume was harvested. The cell suspension was centrifuged and washed twice in fresh synthetic media to remove any remaining glucose, ethanol, and other compounds. Cells were resuspended in synthetic media to a concentration of 14.3 g/L and used to increase cell density in a clean bioreactor by adding the suspension in a stepwise manner. Concentrations were measured in the ranges: 0––247 mM for glucose, 0–500 mM for ethanol, and 0–4.9 g/L for biomass with HPLC and dry-weight determination (Supplementary Table S5.2.1).

### Reference data

#### Biomass

The batch fermentations were sampled every hour, and cell growth was determined by optical density at a wavelength of 660 nm (OD660) using a Libra S11 spectrophotometer (Biochrom, Cambridge, UK). Dry-weight determination was performed by loading 10 mL of culture broth on nitrocellulose membrane filters (pore size, 0.45 μm; Gelman Laboratory, Ann Arbor, MI), drying the filters in a microwave and subsequently weighing the dry biomass (Mettler Toledo, Columbus, USA).

#### HPLC

Sample supernatants of the batch, fed-batch, and single compound samples were analyzed for the corresponding ethanol and glucose concentrations using an Agilent 1260 infinity HPLC (Agilent Technologies, Santa Clara, CA). A BIO-RAD Aminex HPX-87H (300 × 7.8 mm) cation-exchange column (Bio-Rad, Hercules, CA) operated at a temperature of 60 °C and 0.5 g/L H2SO4 was used as eluent with a 0.6 mL/min flow rate. The injection volume was 5 μm, and an Agilent 1260 refractive-index and VWD detector at 214 nm was used for characterization.

### Raman spectroscopy

#### Signal acquisition

A Raman RXN2 analyzer (Kaiser Optical Systems Inc., Ann Arbor, MI) equipped with a 785-nm laser was connected to the bioreactor with a fiber optic cable and bIO-Optic immersion probe to collect spectra over the range of 100–3400 cm^−1^. The immersion probe was mounted through the head plate and sterilized with the bioreactor. Several acquisition settings were assessed to find the optimal acquisition settings which provided a good signal-to-noise ratio while maintaining a high monitoring resolution. An exposure time of 60-s resulted in a detector saturation between 30 and 58% over the full process range. The Raman spectrometer was set to continuously acquire individual 60-s spectra that could be combined into longer measurements after data collection. Datasets of 1, 2, 4, 6, 8, and 10 min matching the timepoint of reference sampling were generated according to a protocol similar to Andre et al. [22]. Initial PLS models were calibrated using all different acquisition lengths, and their prediction performance was evaluated according to the methods described in the “Signal pre-processing and model building” section. Prediction performance remained similar for acquisitions times above 1 min of acquisition time (Supplementary Figure S5.3.1). Therefore, all subsequent models were calibrated with 1-min Raman spectra to maintain a high data resolution during process monitoring. The single compound Raman spectra were acquired by measuring ten individual spectra of 1 min per concentration, and the ten spectra were averaged to obtain noise-free spectra.

#### Signal pre-processing and model building

Spectral pre-processing and model building was performed in MATLAB R2020b (MathWorks, WA), using PLS_Toolbox (v 9.2, Eigenvector Research, WA). The first step of pre-processing was the selecting of the fingerprint region ranging from 450 to 1800 cm^−1^. It was observed that background fluorescence and scattering effects in the spectral dataset increased exponentially towards the lower wavenumber region. Therefore, a basic extended multiplicative scatter correction (EMSC) was chosen using a quadratic term and the average spectra as the regression reference to properly fit and eliminate scattering effects [23]. All datasets were mean-centered before the modeling steps. The PLS models were calibrated by loading the spectra as X-data and the reference measurements per target analyte as the Y-data (HPLC and biomass). Venetian Blinds cross-validation with sevenfold was applied, and the number of latent variables was selected based on the elbow point of the root mean square error of calibration (RMSEC) and cross-validation (RMSECV) scores (Supplementary Figures S5.4.1, S5.4.2). The quantitative validation of the base and supplemented models was performed by applying the models to a validation dataset consisting of an unseen batch fermentation and evaluating the performance based on the RMSEC, RMSECV, and root mean square error of prediction (RMSEP). To compare the performance of the base and supplemented models, relative root mean square errors (rRMSE) were used. An rRMSE based on the interquartile range (IQR) of the calibration dataset (for rRMSEC and rRMSECV) or application dataset (rRMSEP) was chosen (Eq. 1) to reduce the influence of skewed data distributions when assessing model performance:

1 $$rRMSE=\frac{RMSE}{Q3-Q1}\times 100$$

where Q1 is the 25th percentile and Q3 is the 75th percentile of the dataset. This approach ensures the relative error measure is robust to outliers and focuses on the variability within the central portion of the data. The regression coefficient vectors were investigated for qualitative validation of the models and compared to single compound spectra of the target analyte for each model.

## Results and discussion

The impact of combining Raman spectra obtained for batch fermentation processes and single compounds on model specificity and transferability to a related process is determined using a qualitative and quantitative assessment approach. Firstly, the standard modelling approach is performed to obtain a base model. Here, batch fermentation data is used for calibration, and the model is validated using an unseen but similar batch fermentation dataset. This is included to highlight the importance of applying quantitative and qualitative model assessment, as well as for understanding the connection between model specificity and transferability to the fed-batch process. Subsequently, single compound data supplementation is showcased, and the supplemented model performance on both unseen batch and fed-batch data is presented and discussed.

### Quantitative analysis of base model performance for batch data

Partial least square (PLS) models were calibrated using fermentation process data to monitor glucose, ethanol, and biomass concentrations during yeast fermentation with Raman spectroscopy. Three individual base models were built (glucose, ethanol, and biomass) using Raman spectra and reference measurements from three batch cultivations (38 samples, Supplementary Figure S5.1.1). These base models were subsequently validated on data of one unseen batch cultivation (13 samples). The resulting model statistics and performances are shown in Table 1.

**Table 1:** Overview of statistics for the quantitative assessment of the base models for glucose, ethanol, and biomass

Parameter	Calibration range	RMSEC	RMSECV	RMSEP	rRMSEP	Latent variables
Glucose	0–120.78 mM	1.67 mM	1.83 mM	1.46 mM	2.07%	2
Ethanol	0–172.86 mM	3.67 mM	4.34 mM	4.36 mM	3.57%	2
Biomass	0.10–3.23 g/L	0.07 g/L	0.08 g/L	0.11 g/L	5.38%	2

The quantitative analysis for all three models displays relative root mean square error of prediction (rRMSEP) values below 5% for the glucose and ethanol model and 5.38% for the biomass model. The RMSEP and RMSECV values of each model are close together, suggesting over- or under-fitting of the calibration data does not occur, and that the models perform well on unseen data. This was expected as the unseen batch was operated under identical conditions as the batches used for calibration. This means that quantitative assessment of base model performance according to common practice in literature indicates that the models are well calibrated for quantification during batch fermentation [10].

### Base model performance on fed-batch data

**Fig. 1:**
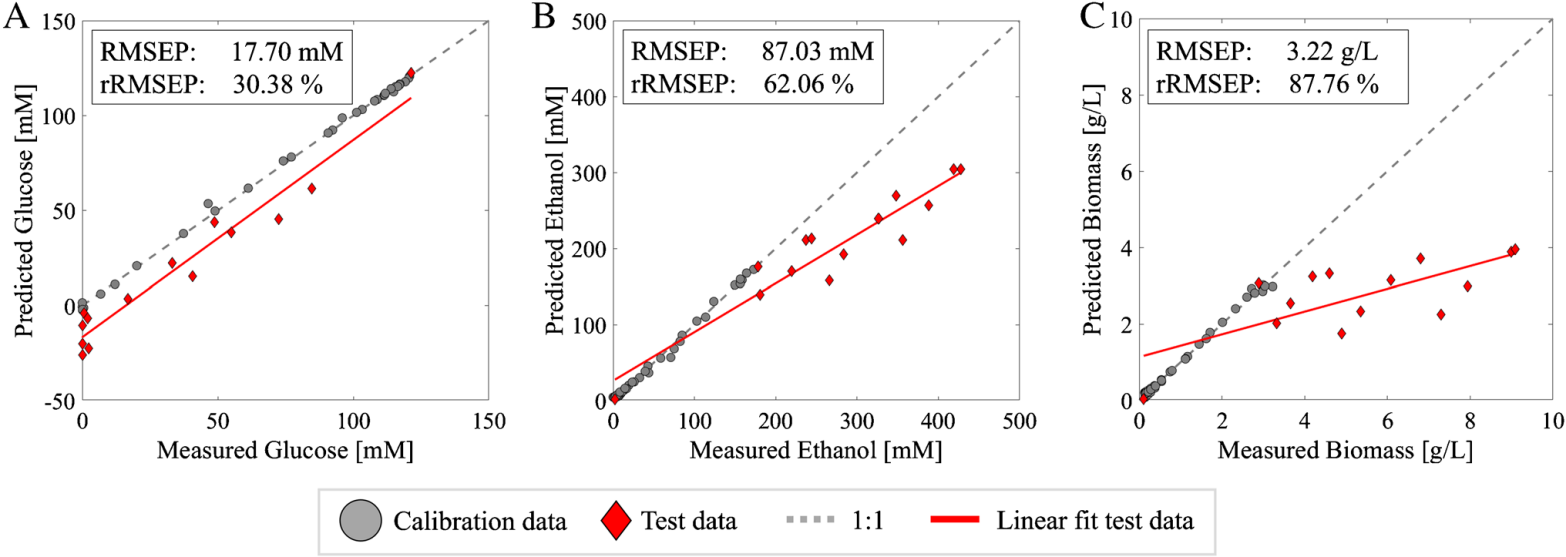
Measured (*x*-axis) versus predicted (*y*-axis) concentration plots of **A** glucose in mM, **B** ethanol in mM, and **C** biomass in g/L of base models applied to spectra obtained during fed-batch cultivation. The fed-batch data is shown as red diamonds, the base calibration data as gray circles, the 1:1 fit as the grey dotted line, and the data fit as the red line. The RMSEP and rRMSEP of each model applied to the fed-batch are shown in the boxes

In this section, we simulate a model transfer case by transferring the validated base models to a yeast fed-batch fermentation. Through this transfer, we evaluate the effectiveness of the base models on a related process containing the same process analytes, but with altered inter-compound ratios (all three analytes) extended concentration ranges (ethanol, biomass). The model performance in shown in the form of measured versus predicted concentration plots in Fig. 1A–C, along with the statics for quantitative assessment of the base model performance on unseen fed-batch process data in Fig. 1D.

Bolus feeding of glucose during the fed-batch process disrupted the ability of all three base models to accurately quantify their target analytes. This is represented by the increased RMSEP and rRMSEP values shown in Fig. 1. Figure 1A shows that the glucose sample at 120 mM is predicted well by the base model, which was taken directly after inoculation and therefore considered highly similar to the batch data. However, new spectral variations in subsequent fed-batch samples caused an underestimation of the glucose concentrations. The RMSEP of the glucose model is 12 times higher when applied to the fed-batch when compared to the batch fermentation, despite the concentrations being in the same range. This highlights the lack of model specificity of glucose and the model not being able to deal with the new ratios between the compounds. A comparable underestimation is seen for ethanol and biomass (Fig. 1B and C, respectively), where the base models were forced to extrapolate due to the extended concentration ranges. For the ethanol and biomass models, the three feeding moments are visible as individual groups of samples, where the predictions move further from the measured values with each glucose addition. Next to extrapolation, the decrease in prediction performance could result from the exponential process and hourly sampling interval of the batch calibration dataset. This led to an uneven sample distribution and a possible accuracy bias towards early-stage batch conditions, which contains high glucose, low ethanol, and low biomass concentrations, and therefore underestimating ethanol and biomass concentrations.

### Qualitative assessment of base model

**Fig. 2:**
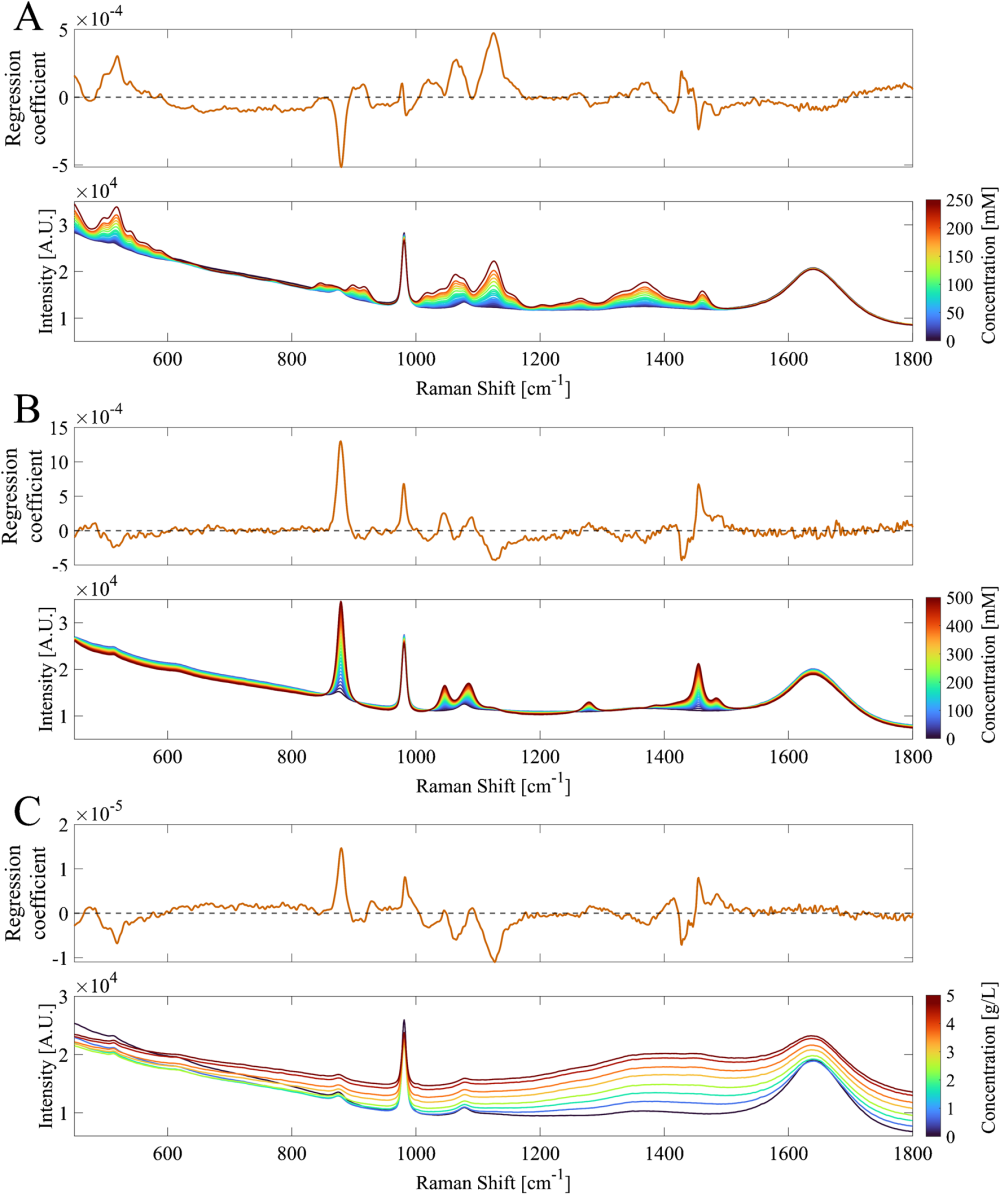
The regression coefficient vectors (top) of the **A** glucose in mM, **B** ethanol in mM, and **C** biomass in g/L base models. Each regression vector coefficient is juxtaposed with a concentration range of unprocessed single compound spectra of the models target parameter (bottom). The heatmap of the concentration range shows a low analyte concentration in blue and a high analyte concentration in red

Extrapolation and skewed sample distributions may not be the sole reasons for the decreased performance. PLS models assume a linear relation between the signal response and analyte concentration, and a robust model can sometimes extrapolate predictions outside of its calibration range with moderate accuracy, assuming that the relationships between the variables remain consistent [24]. As no new analytes were introduced in the fed-batch, the new spectral variations and high RMSEPs are most likely related to the different proportions between the parameters as a result of bolus feeding. Furthermore, the lack of extrapolation capabilities indicates that the obtained base models are not specific to their target analyte. Calibration with batch data led to cross-correlations in the base models, supported by the Pearson coefficients above 0.980 between all analytes (Supplementary Figure S5.5.1). A standard quantitative assessment statistic such as RMSEP does not indicate the specificity of each model to its target. The specificity of each base model can be visually inspected by comparing the regression coefficient values for each wavenumber with the single compound spectra of the targets [25]. The regression coefficient vector (RCV) of each base model is compared to single compound spectra of the target analyte in Fig. 2.

The regression coefficient vector of the glucose base model (Fig. 2A) displays a positive correlation to known spectral markers for glucose, such as the C_2_-C_1_-O_1_ bending at 517 cm^−1^ and the C-O–H bending at 1125 cm^−1^ [26]. However, negative correlations to ethanol peaks are also observed, such as the C–C stretching peak at 879 cm^−1^ [27]. It should be noted that negative regression coefficients do not necessarily represent negative correlations, as negative regression coefficients could result from the mathematical constraints of PLS when peaks overlap [25]. Based on single compound spectra of the three main analytes, the 879 cm^−1^ peak is mostly free from glucose and biomass peaks. Thus, it can be concluded that glucose quantification is coupled to the 879 cm^−1^ ethanol peak, causing underprediction of glucose concentrations for the higher ethanol concentrations during the fed-batch fermentation. The regression coefficient of the ethanol model (Fig. 2B) is positively correlated to ethanol characteristic spectral markers, such as C–C stretching, C-O stretching, and CH_3_ rocking at 879 cm^−1^, 1046 cm^−1^, and 1084 cm^−1^, respectively [27]. However, it also includes negative correlations to known glucose peaks (C_2_-C_1_-O_1_ bending at 517 cm^−1^, C-O–H bending at 1125 cm^−1^). Similarly to the glucose base model, the correlations to glucose-specific peaks cause the ethanol base model to underpredict ethanol concentrations. Each time the glucose concentration was increased through bolus feeding, the underprediction of ethanol concentration increased (Fig. 1B). The regression coefficient of the biomass base model (Fig. 2C) is highly similar in structure to the ethanol base model (Fig. 2B), indicating that biomass prediction is based on the decrease of glucose and increase of ethanol concentration. This is a result of the high cross-correlation between biomass and both ethanol and glucose in the calibration dataset, reflected by a *R*^2^ of 0.992 and 0.988, respectively (Supplementary Figure S5.5.1).

The base models were quantitatively validated by getting low RMSEP values when applied to the unseen batch data (Table 1). However, qualitative assessment showed that the base models are heavily dependent on variations not related to the target analyte, but rather reflect batch process evolution, which is a result of maximizing the covariance between *X* and *Y* with an implicit modelling technique. Every biological process has inherent cross-correlations, and if calibration datasets are not constructed appropriately, these cross-correlations are integrated into the analyte quantification model. To extend the applicability of process data beyond the original process, target specificity needs to be assessed before model transfer. The lack of qualitative assessment may not only pose a risk when moving to different modes of operation, and model performance may also be compromised when there is a deviation in one of the correlated analytes while running a similar process. Events such as a deviation in inoculation cell density or ethanol carryover from the preculture to a bioreactor can all introduce errors, as a change in one analyte concentration directly affects the prediction of the other two compounds. Although the challenges shown with the models in this work are of a specific case where only exponential growth phase data was used for calibration, the issues related to non-specificity in Raman model development for upstream bioprocesses have been reported on before [15, 18].

### Impact of single compound spectra data supplementation

**Fig. 3:**
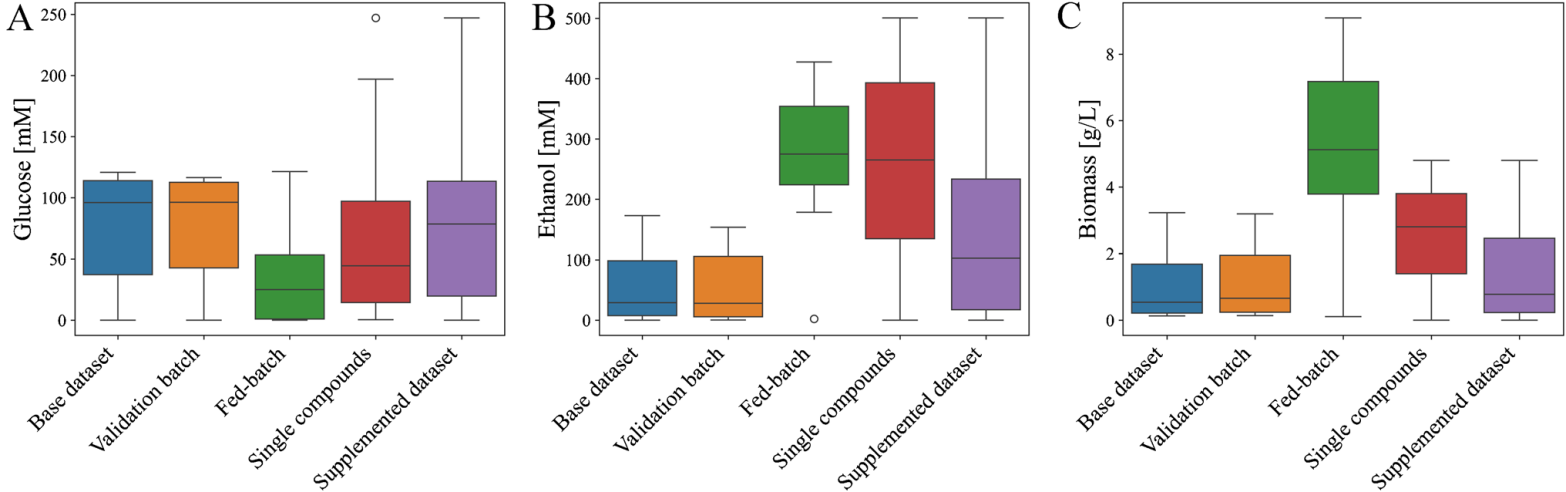
Sample distributions for **A** glucose in mM, **B** ethanol in mM, and **C** biomass in g/L for each of the datasets: base dataset, validation batch, fed-batch, single compounds, and the supplemented dataset

The decrease in base model performance when transferred from batch data to fed-batch data was due to (1) redistribution of ratios between the target compounds and (2) extrapolation from calibration ranges. This indicated a lack of target analyte specificity in the base model and prediction performance on the fed-batch process should increase when this limitation is overcome. The standard approach is to run one or more fed-batch fermentations and collect new in-line and reference data, which can be either used to train a specific fed-batch fermentation model or combined with the existing batch fermentation data. However, additional fermentation runs would require significant time and effort, thus leading to a delayed ability to monitor a new but related process. As a faster and less labor-intensive alternative, we propose model transfer from a batch fermentation to a fed-batch fermentation by including solely single compound spectra to the batch fermentation calibration dataset. This means that the resulting calibration dataset is a combination of process data and single compound spectra. The single compound data supplementation aims to extend the calibration ranges for the ethanol and biomass models and to improve the specificity of all three models towards its analytical target. An overview of sample distributions of the different datasets is shown in Fig. 3.

The supplementary spectra were collected using the same 60-s acquisition time as during the fermentations. However, constant concentration conditions enabled the acquisition of multiple spectra per concentration point. For each point, ten 60-s measurements were averaged into one spectrum, resulting in high-quality smooth spectra with detector saturations identical to those observed during the fermentations. The new glucose calibration range was extended from 0–120.78 mM to 0–247.08 mM, with an increased sample density in the low concentration range (Fig. 3A). The calibration range of ethanol was extended from 0–172.86 mM to 0–500.68 mM to remove the need of extrapolation outside of the calibration data (Fig. 3B). Acquisition of single biomass spectra resulted in an extended calibration range from 3.23 to 4.81 g/L (Fig. 3C). Due to technical difficulties, not all fed-batch biomass concentrations were reached. Figure 3 shows an increase in sample density in the low glucose and high ethanol concentration ranges, but the data supplementation did not shift the mean concentrations to center of the calibration range. This is important as a mean near the sample distribution center indicates that the samples are properly distributed to prevent accuracy biases to specific concentration regions. The three base models were re-calibrated with the supplemented datasets, referred to as supplemented models. The performance of the supplemented models was evaluated with the fed-batch dataset, shown in Table 2, as well as the unseen batch data (Supplementary Table 5.6.1) to show maintained prediction accuracy for the original process.

**Table 2:** Overview of statistics for the supplemented models for glucose, ethanol, and biomass when applied to the fed-batch data. The last column shows the improvement in rRMSEP of the supplemented model over the base model for the fed-batch data

Model target	Calibration range	RMSEC	RMSECV	RMSEP	rRMSEP	Latent variables	rRMSEP base model	rRMSEP improvement
Glucose	0–247.08 mM	3.05 mM	3.39 mM	3.06 mM	5.25%	2	30.38%	82.72%
Ethanol	0–00.68 mM	8.02 mM	8.14 mM	8.65 mM	6.17%	2	62.06%	90.05%
Biomass	0.10–4.81 g/L	0.18 g/L	0.24 g/L	0.99 g/L	26.98%	3	87.76%	69.26%

The number of latent variables for the biomass supplemented model increased to 3, based on the RMSEC vs RMSECV graphs (Supplementary Figure S5.4.2). The predictive performance of all supplemented models on the fed-batch data increased compared to the base models, reflected by an 82.72%, 90.05%, and 69.26% rRMSEP decrease for glucose, ethanol, and biomass, respectively. The performance of the glucose and ethanol supplemented models is sufficient for accurate monitoring, as the rRMSEP values were close to 5%. The rRMSEP of the biomass supplemented model was 26.98% and only gives an approximation of the biomass concentration. The biomass supplemented model showed increased values for rRMSEC (from 4.45 to 7.85%), rRMSECV (from 6.99 to 10.47%), and rRMSEP (from 5.38 to 8.32%) when applied to the batch validation dataset, meaning that supplementation and improved fed-batch performance came at the cost of batch prediction accuracy and led to a more complex model (from 2 to 3 latent variables) (Supplementary Table S5.6.2).

**Fig. 4:**
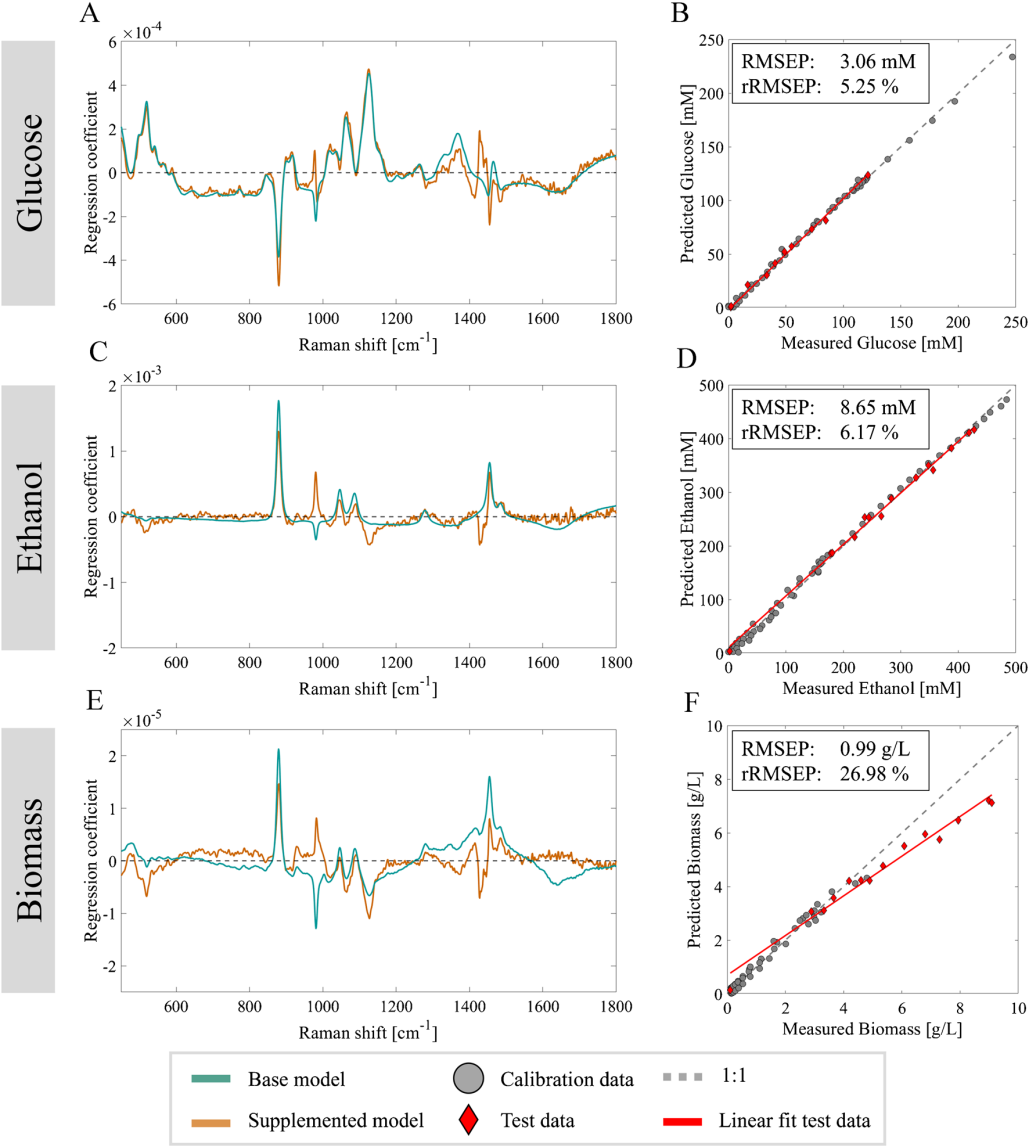
Regression coefficient vectors of the base model (cyan), supplemented model (orange), and the measured versus predicted plots of the supplemented models on the fed-batch dataset with the calibration data (grey) and fed-batch data (red) for glucose (**A**, **B**), ethanol (**C**, **D**), and biomass (**E**, **F**)

For glucose and ethanol, data supplementation resulted in improved performance on the fed-batch cultivation while maintaining similar rRMSEC and rRMSECV values and performance on the validation batch. The decreased RMSEP, improved sample distribution for glucose, and the broader calibration range for ethanol successfully extended the applicability of the models, without compromising on the prediction performance on the original batch validation set. In addition to quantitative validation of the supplemented models, a qualitative assessment using regression vectors was performed to assess the impact on model specificity toward the target analytes (Fig. 4A, C, and E). The corresponding measured versus predicted plots of the supplemented models applied to the fed-batch data are shown in Fig. 4B, D, and F.

The noise in all regression coefficients was reduced by data supplementation with single compound spectra. The coefficients of the glucose supplemented model (Fig. 4A) show decreased dependency on the major ethanol peak at 879 cm^−1^, but the correlation was not completely removed. In addition, the glucose supplemented model showed a decrease in the magnitude of the negative regression coefficients around 1452 cm^−1^. Ethanol has a strong peak at 1455 cm^−1^ belonging to the asymmetric deformation of CH_3_, which overlaps with glucose peaks in the same region. The glucose supplemented model corrects for this overlap by assigning negative regression coefficients to the ethanol peak to prevent an additive effect of these peaks and overestimation of the concentration [25]. For the glucose base model, these negative regression coefficients were stronger due to the cross-correlation to the ethanol peak at 1455 cm^−1^. Figure 4C shows that single compound samples had high leverage on the ethanol supplemented model as the regression coefficient vector closely represents the ethanol single compound spectra (Fig. 2B). Although specificity of the model has increased, the current regression coefficient vector does not extensively compensate for overlapping glucose peaks. This could lead to possible overestimation of ethanol concentrations in situations where the concentrations of both ethanol and glucose are high, due to overlapping peaks in the 1000–1150 cm^−1^ and 1400–1500 cm^−1^ regions.

The biomass supplemented model underpredicts the actual biomass concentrations, suggesting there still is a dependency on non-target related peaks. The single compound spectra for biomass leveraged the model to assign more weights to the 1300–1500 cm^−1^ range where the single compound spectra for biomass displayed a baseline increase in the fingerprint region located between 1200 and 1600 cm^−1^ for higher biomass concentrations (Fig. 2C). However, the regression coefficients are not specific enough for predictions independent of glucose and ethanol peaks. During the batch fermentations, a non-linear signal extinction over the fingerprint region was observed, contradictory to the ascending baseline for single compound biomass spectra (Fig. 2C). Iversen observed a similar non-uniform signal extinction with increasing yeast biomass during fermentation and explained the effect to be caused by Lorenz-Mie scattering from the cell as particles [28]. It is unknown what caused the baseline increase in the single compound biomass spectra. At this point in time, we cannot fully exclude the impact of increased background fluorescence by media compounds which was used as measurement matrix, (decreased) cell culture viability, or possible leakage of cell contents. The yeast biomass used for these measurements was harvested after glucose depletion during batch fermentation and then washed and resuspended in clean synthetic media devoid of substrates and metabolites. Supernatant analysis with HPLC of samples taken during the biomass measurements showed no presence of glucose, ethanol, or other metabolites. While the consistent use of synthetic media maintained osmotic consistency, the extended depletion of substrates over the ~ 2-h harvest and measurement procedure may have induced stress in the cells. More measurements of single yeast suspensions are required to gain knowledge on the spectral contribution of cells.

After evaluating the performance of the base models on the fed-batch data, it was observed that the decrease in performance was due to the redistribution of ratios between target compounds and the extrapolation beyond the original calibration ranges. Since PLS models assume a linear relationship between compound concentration and signal intensity, moderate prediction accuracy can be expected when the model extrapolates. It is therefore crucial that the models are first made robust against cross-correlations between process compounds before considering any calibration range extensions. Had the original base models been calibrated using batch process data with higher concentration ranges for all compounds, the fed-batch concentrations would have fallen within those ranges. However, the batch data would still contain strong cross-correlations between compounds, and application to the fed-batch where the ratios between these compounds change would still be problematic. Thus, addressing cross-correlation issues is key to developing more robust models, which provide a stronger foundation for extending the calibration range.

Qualitative assessment show that PLS models calibrated with process specific Raman spectra can be improved in terms of target specificity and quantification range by supplementing calibration datasets with single compound spectra. Supplementation with single compound spectra obtained in bioreactors offers a simple method for calibration dataset improvement without the need for extensive experimental designs and sample preparation, while maintaining process conditions such as temperature, stirring, and sparging. Moreover, increased model specificity results in the ability to transfer a base model beyond the original process it was trained for. Although the single compound supplementation successfully reduced cross-correlations in the calibration datasets, a next step could be to investigate solutions to efficiently generate mixture spectra which could disrupt the cross-correlations more efficiently. The observed discrepancy between single biomass spectra and process data and the lack of knowledge on clear spectral markers for yeast biomass in literature indicate that extensive studies on the individual effect of biomass on Raman spectra should be performed. More research on the spectral signals of cell density and viability will aid in developing specific biomass quantification models independent of substrate and product peaks.

## Conclusion

Partial least square (PLS) quantification models for Raman spectroscopy-based real-time fermentation monitoring are commonly calibrated with process data. However, fermentation processes have inherent cross-correlations leading to process specific models which do not transfer to related processes. Model calibration with highly cross-correlated data leads to prediction co-dependencies, and standardly reported quantitative model validation, using metrics such as (r)RMSEP, does not guarantee model quality. Model specificity and spectral selectivity is essential for model robustness and should be evaluated during model calibration by investigating model statistics, such as the regression coefficients. Our approach of data supplementation with single compound spectra of the quantification target can expand calibration ranges, re-distribute a model’s weights, and improve specificity to the relevant spectral markers. This extends the applicability of existing models to related processes, without the need of collecting new process data. We demonstrated this by adjusting base models calibrated on batch fermentation data, allowing transfer to a fed-batch mode of operation. This is represented by an rRMSEP improvement of 82.72%, 90.05%, and 69.26% for glucose, ethanol, and biomass, respectively, leading to the absolute RMSEPs of 3.06 mM, 8.65 mM, and 0.99 g/L. Using single compound data spectra supplementation offers a fast and simple alternative to full model re-calibration, spiked samples integration, or extensive DoE approaches. Approaches like these can speed up the implementation and application of real-time monitoring with Raman spectroscopy and thereby aid to early process monitoring and efficient process development.

## Supplementary Information

Below is the link to the electronic supplementary material.

Supplementary file1 (DOCX 1112 KB)

## References

[1] O’Mara P, Farrell A, Bones J, Twomey K. Staying alive! Sensors used for monitoring cell health in bioreactors. Talanta. 2018;176:130–9. 10.1016/j.talanta.2017.07.088. 28917732 10.1016/j.talanta.2017.07.088

[2] FDA. Guidance for industry: PAT—A framework for innovative pharmaceutical development, manufacturing, and quality assurance. Rockville, MD: Food and Drug Administration; 2004.

[3] Vasilescu A, Fanjul-Bolado P, Titoiu AM, Porumb R, Epure P. Progress in electrochemical (bio) sensors for monitoring wine production. Chemosensors. 2019;7(4):66. 10.3390/chemosensors7040066.

[4] Bergin A, Carvell J, Butler M. Applications of bio-capacitance to cell culture manufacturing. Biotechnol Adv. 2022;61:108048. 10.1016/j.biotechadv.2022.108048. 36208846 10.1016/j.biotechadv.2022.108048

[5] Wasalathanthri DP, Rehmann MS, Song Y, Gu Y, Mi L, Shao C, Chemmalil L, Lee J, Ghose S, Borys MC. Technology outlook for real-time quality attribute and process parameter monitoring in biopharmaceutical development—a review. Biotechnol Bioeng. 2020;117(10):3182–98. 10.1002/bit.27461. 32946122 10.1002/bit.27461

[6] Rathore A, Bhambure R, Ghare V. Process analytical technology (PAT) for biopharmaceutical products. Anal Bioanal Chem. 2010;398(1):137–54. 10.1007/s00216-010-3781-x. 20480150 10.1007/s00216-010-3781-x

[7] Lourenço N, Lopes J, Almeida C, Sarraguça M, Pinheiro H. Bioreactor monitoring with spectroscopy and chemometrics: a review. Anal Bioanal Chem. 2012;404(4):1211–37. 10.1007/s00216-012-6073-9. 22644146 10.1007/s00216-012-6073-9

[8] Hirsch E, Pataki H, Domján J, Farkas A, Vass P, Fehér C, Barta Z, Nagy ZK, Marosi GJ, Csontos I. Inline noninvasive Raman monitoring and feedback control of glucose concentration during ethanol fermentation. Biotechnol Prog. 2019;35(5):e2848. 10.1002/btpr.2848. 31115976 10.1002/btpr.2848

[9] Webster TA, Hadley BC, Hilliard W, Jaques C, Mason C. Development of generic Raman models for a GS-KOTM CHO platform process. Biotechnol Prog. 2018;34(3):730–7. 10.1002/btpr.2633. 29603893 10.1002/btpr.2633

[10] Esmonde-White KA, Cuellar M, Lewis IR. The role of Raman spectroscopy in biopharmaceuticals from development to manufacturing. Anal Bioanal Chem. 2021;414:1–23. 10.1007/s00216-021-03727-4. 10.1007/s00216-021-03727-4 PMC8724084 34668998

[11] Zavala-Ortiz DA, et al. Comparison of partial least square, artificial neural network, and support vector regressions for real-time monitoring of CHO cell culture processes using in situ near-infrared spectroscopy. Biotechnol Bioeng. 2022;119(2):535–49. 10.1002/bit.27997. 34821379 10.1002/bit.27997

[12] Rafferty C, Johnson K, O’Mahony J, Burgoyne B, Rea R, Balss KM. Analysis of chemometric models applied to Raman spectroscopy for monitoring key metabolites of cell culture. Biotechnol Prog. 2020;36(4):e2977. 10.1002/btpr.2977. 32012476 10.1002/btpr.2977

[13] Kozma B, Salgó A, Gergely S. Comparison of multivariate data analysis techniques to improve glucose concentration prediction in mammalian cell cultivations by Raman spectroscopy. J Pharm Biomed Anal. 2018;158:269–79. 10.1016/j.jpba.2018.06.005. 29894949 10.1016/j.jpba.2018.06.005

[14] Wold S, Sjöström M, Eriksson L. PLS-regression: a basic tool of chemometrics. Chemom Intell Lab Syst. 2001;58(2):109–30. 10.1016/S0169-7439(01)00155-1.

[15] Santos RM, Kessler JM, Salou P, Menezes JC, Peinado A. Monitoring mAb cultivations with in-situ Raman spectroscopy: the influence of spectral selectivity on calibration models and industrial use as reliable PAT tool. Biotechnol Prog. 2018;34(3):659–70. 10.1002/btpr.2635. 29603907 10.1002/btpr.2635

[16] André S, Lagresle S, Da Sliva A, Heimendinger P, Hannas Z, Calvosa É, Duponchel L. Developing global regression models for metabolite concentration prediction regardless of cell line. Biotechnol Bioeng. 2017;114(11):2550–9. 10.1002/bit.26368. 28667738 10.1002/bit.26368

[17] Domján J, Fricska A, Madarász L, Gyürkés M, Köte Á, Farkas A, Vass P, Fehér C, Horváth B, Könczöl K. Raman-based dynamic feeding strategies using real-time glucose concentration monitoring system during adalimumab producing CHO cell cultivation. Biotechnol Prog. 2020;36(6):e3052. 10.1002/btpr.3052. 32692473 10.1002/btpr.3052

[18] Romann P, Kolar J, Tobler D, Herwig C, Bielser JM, Villiger TK. Advancing Raman model calibration for perfusion bioprocesses using spiked harvest libraries. Biotechnol J. 2022;17:2200184. 10.1002/biot.202200184. 10.1002/biot.202200184 35900328

[19] Webster TA, Hadley BC, Dickson M, Hodgkins J, Olin M, Wolnick N, Armstrong J, Mason C, Downey B. Automated Raman feed-back control of multiple supplemental feeds to enable an intensified high inoculation density fed-batch platform process. Bioprocess Biosyst Eng. 2023;46:1457–70. 10.1007/s00449-023-02912-2. 37633861 10.1007/s00449-023-02912-2

[20] Nijkamp JF, van den Broek M, Datema E, de Kok S, Bosman L, Luttik MA, Daran-Lapujade P, Vongsangnak W, Nielsen J, Heijne WH. De novo sequencing, assembly and analysis of the genome of the laboratory strain Saccharomyces cerevisiae CEN. PK113–7D, a model for modern industrial biotechnology. Microbial Cell Factories. 2012;11(1):1–17. 10.1186/1475-2859-11-36. 22448915 10.1186/1475-2859-11-36 PMC3364882

[21] Verduyn C, et al. Effect of benzoic acid on metabolic fluxes in yeasts: a continuous-culture study on the regulation of respiration and alcoholic fermentation. Yeast. 1992;8(7):501–17. 10.1002/yea.320080703. 1523884 10.1002/yea.320080703

[22] André S, Lagresle S, Hannas Z, Calvosa É, Duponchel L. Mammalian cell culture monitoring using in situ spectroscopy: is your method really optimised? Biotechnol Prog. 2017;33(2):308–16. 10.1002/btpr.2430. 28019710 10.1002/btpr.2430

[23] Martyna A, Menżyk A, Damin A, Michalska A, Martra G, Alladio E, Zadora G. Improving discrimination of Raman spectra by optimising preprocessing strategies on the basis of the ability to refine the relationship between variance components. Chemom Intell Lab Syst. 2020;202:104029. 10.1016/j.chemolab.2020.104029.

[24] Balabin RM, Smirnov SV. Interpolation and extrapolation problems of multivariate regression in analytical chemistry: benchmarking the robustness on near-infrared (NIR) spectroscopy data. Analyst. 2012;137(7):1604–10. 10.1039/c2an15972d. 22337290 10.1039/c2an15972d

[25] Seasholtz MB, Kowalski BR. Qualitative information from multivariate calibration models. Appl Spectrosc. 1990;44(8):1337–48. 10.1366/000370290789619478.

[26] Dudek M, Zajac G, Szafraniec E, Wiercigroch E, Tott S, Malek K, Kaczor A, Baranska M. Raman optical activity and Raman spectroscopy of carbohydrates in solution. Spectrochim Acta Part A Mol Biomol Spectrosc. 2019;206:597–612. 10.1016/j.saa.2018.08.017. 10.1016/j.saa.2018.08.017 30196153

[27] Boyaci IH, Genis HE, Guven B, Tamer U, Alper N. A novel method for quantification of ethanol and methanol in distilled alcoholic beverages using Raman spectroscopy. J Raman Spectrosc. 2012;43(8):1171–6. 10.1002/jrs.3159.

[28] Iversen JA, Berg RW, Ahring BK. Quantitative monitoring of yeast fermentation using Raman spectroscopy. Anal Bioanal Chem. 2014;406(20):4911–9. 10.1007/s00216-014-7897-2. 24996999 10.1007/s00216-014-7897-2

