# Supplementary material for: Single compound data supplementation to enhance transferability of fermentation specific Raman spectroscopy models
Source: Anal Bioanal Chem. 2025 Feb 6;417(9):1873–84. doi: 10.1007/s00216-025-05768-5 (PMC11914363; doi:10.1007/s00216-025-05768-5)
Supplement: Supplementary file 1 — Supplementary file1 (DOCX 1112 KB) [file 216_2025_5768_MOESM1_ESM.docx]

Single compound data supplementation to enhance transferability of ferementation specific Raman spectroscopy models

Supplementary figures

Maarten Klaverdijk^1^, Marcel Ottens^1^, Marieke Klijn^1*^

^1^ Department of Biotechnology, Delft University of Technology, Van der Maasweg 9, Delft, 2629 HZ, The Netherlands

* Corresponding author. Tel: +31 15 27 81280. E-mail address: m.e.klijn@tudelft.nl

**5. Supplementary figures**

**5.1 Fermentation time evolution graphs**


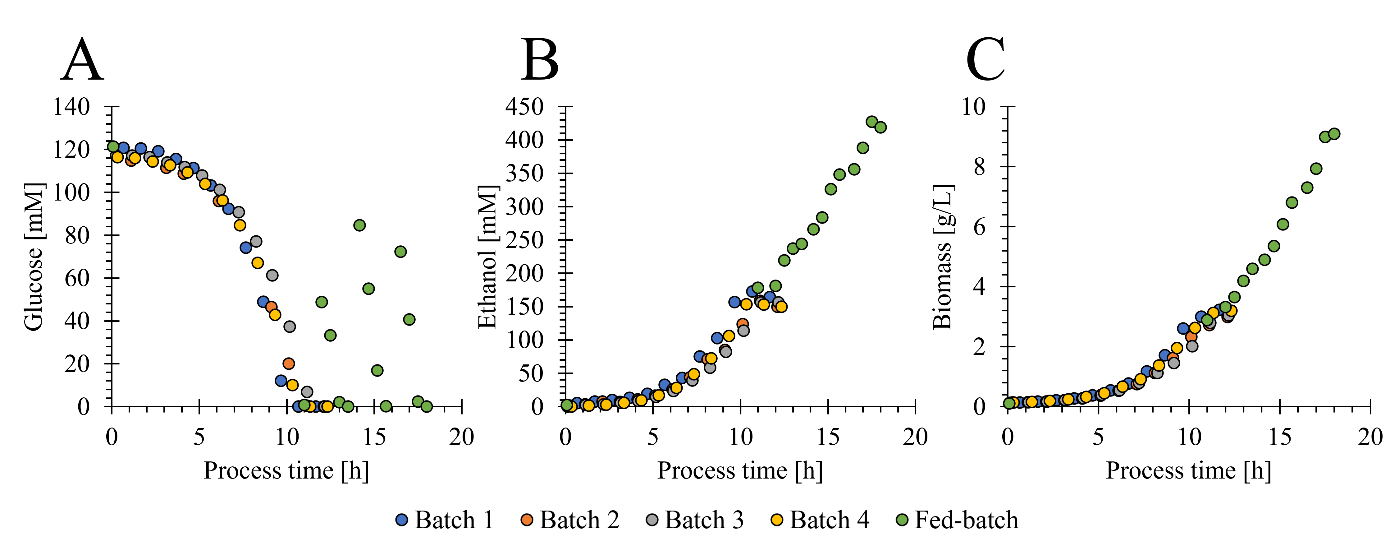


**Figure 5.1.1:** The concentrations of glucose (A), ethanol (B), and biomass (C) over process time for the calibration batches (Batch 1-3, blue, orange, and gray), validation batch (batch 4, yellow), and the fed-batch test dataset (green).

**5.2 Overview of pure component measurement concentrations**

**Table 5.2.1:** Concentrations of the supplementary measured samples for glucose, ethanol, and biomass.

| Sample | Glucose [mM] | Ethanol [mM] | Biomass [g/L] |
| --- | --- | --- | --- |
| 1 | 0.45 | 0.00 | 0.00 |
| 2 | 0.87 | 17.46 | 0.80 |
| 3 | 2.33 | 36.03 | 1.60 |
| 4 | 4.37 | 55.05 | 2.50 |
| 5 | 7.22 | 74.35 | 3.10 |
| 6 | 9.64 | 91.12 | 3.60 |
| 7 | 14.45 | 109.82 | 4.40 |
| 8 | 19.53 | 123.72 | 4.80 |
| 9 | 24.51 | 145.42 | - |
| 10 | 29.50 | 161.07 | - |
| 11 | 33.88 | 179.52 | - |
| 12 | 39.00 | 198.80 | - |
| 13 | 44.51 | 216.16 | - |
| 14 | 49.32 | 233.44 | - |
| 15 | 59.14 | 248.02 | - |
| 16 | 68.79 | 265.12 | - |
| 17 | 78.48 | 282.14 | - |
| 18 | 87.84 | 299.38 | - |
| 19 | 97.11 | 315.18 | - |
| 20 | 112.80 | 332.94 | - |
| 21 | 138.57 | 347.86 | - |
| 22 | 157.54 | 367.24 | - |
| 23 | 177.43 | 386.56 | - |
| 24 | 196.91 | 399.72 | - |
| 25 | 247.08 | 416.08 | - |
| 26 | - | 430.80 | - |
| 27 | - | 444.84 | - |
| 28 | - | 455.32 | - |
| 29 | - | 474.44 | - |
| 30 | - | 484.08 | - |
| 31 | - | 500.68 | - |

**5.3 Influence of acquisition time on model accuracy**


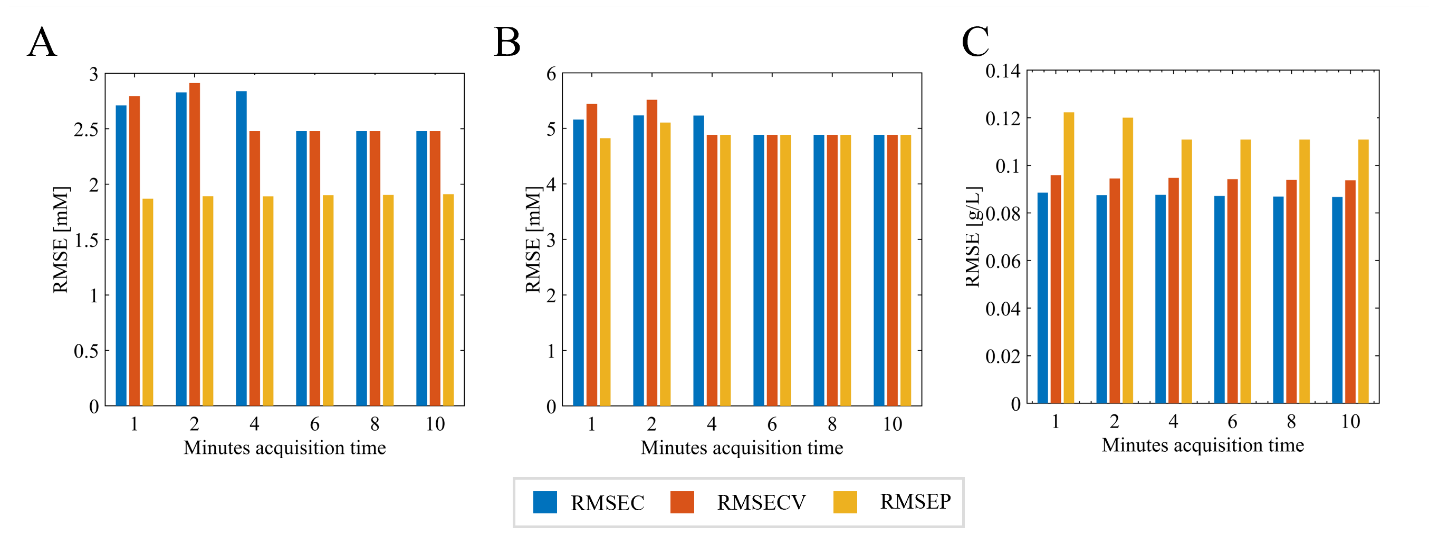


**Figure 5.3.1:** The performance for different acquisition times in minutes shown in root mean square errors of calibration (RMSEC), cross-validation (RMSECV), and prediction (RMSEP) for the base model calibration (Batch 1-3) and application to the batch validation dataset (Batch 4) of glucose (A), ethanol (B), and biomass (C).

**5.4 RMSEC vs RMSECV plots for base and supplemented models**


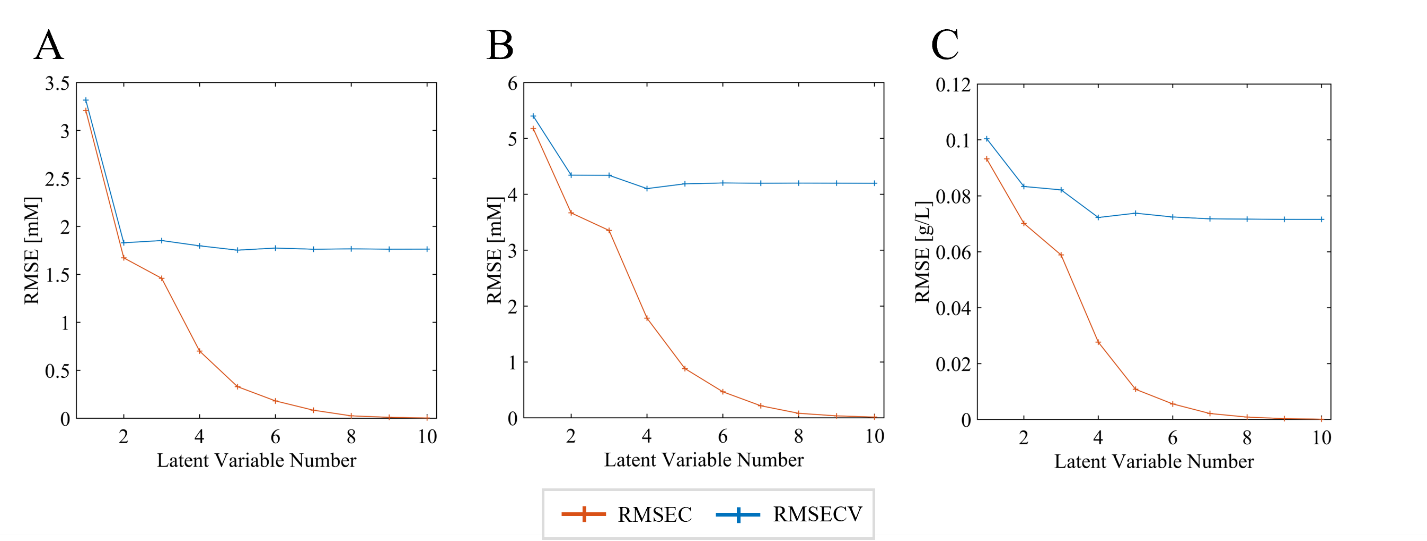


**Figure 5.4.1:** Elbow plots showing the root mean square error of calibration (RMSEC) and cross-validation (RMSECV) (x-axis) vs latent variables (y-axis) to select the number of latent variables for the base models of glucose (A), ethanol (B), and biomass (C).


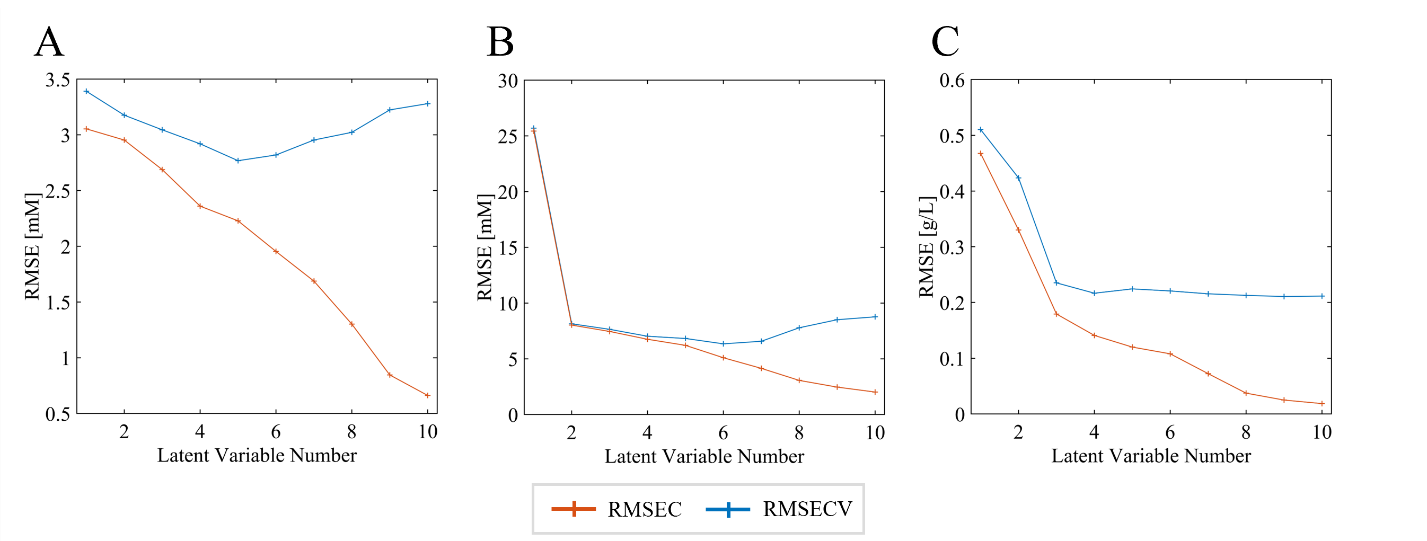


**Figure 5.4.2:** Elbow plots showing the root mean square error of calibration (RMSEC) and cross-validation (RMSECV) (x-axis) vs latent variables (y-axis) to select the number of latent variables for the supplemented models of glucose (A), ethanol (B), and biomass (C).

**5.5 Cross-correlations between process compounds in the base and supplemented datasets**


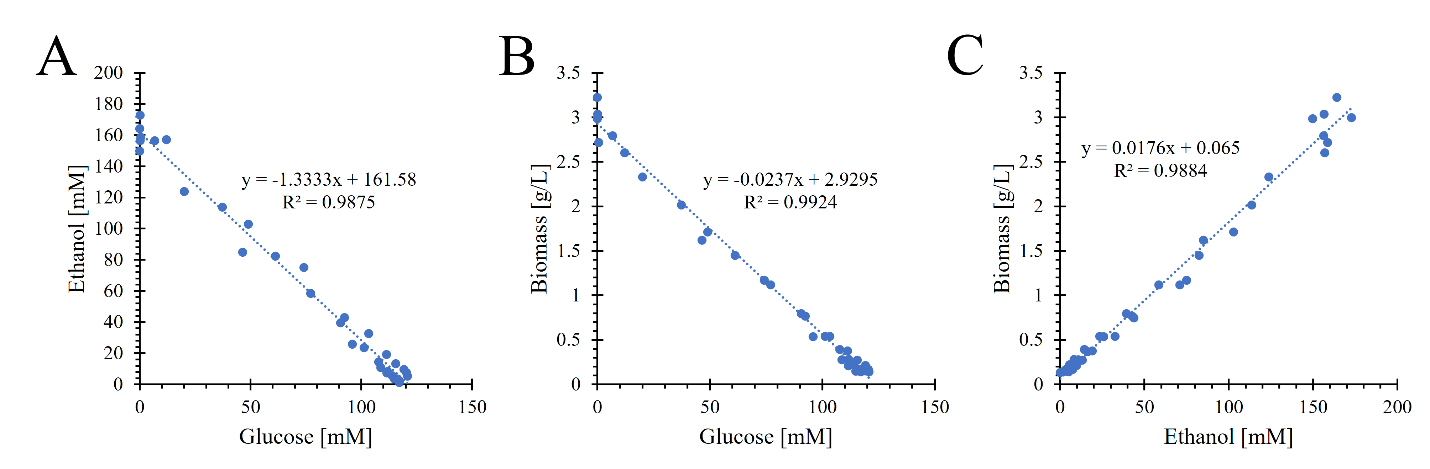


**Figure 5.5.1:** The cross-correlations in the base calibration dataset represented as glucose versus ethanol (A), glucose versus biomass (B), and ethanol versus biomass (C).


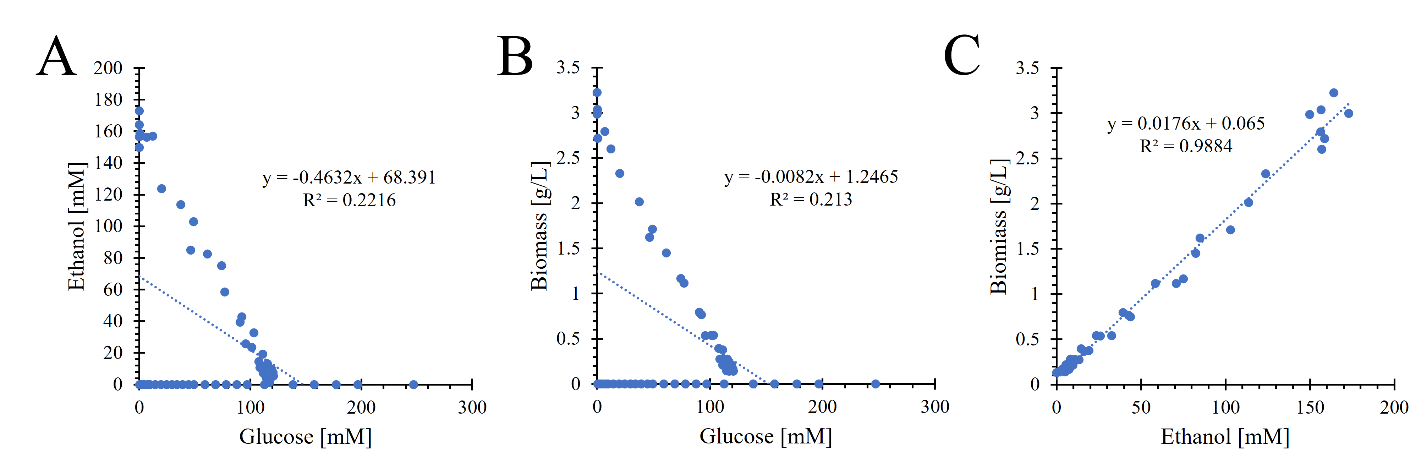


**Figure 5.5.2:** The cross-correlations in the glucose supplemented calibration dataset represented as glucose versus ethanol (A), glucose versus biomass (B), and ethanol versus biomass (C).


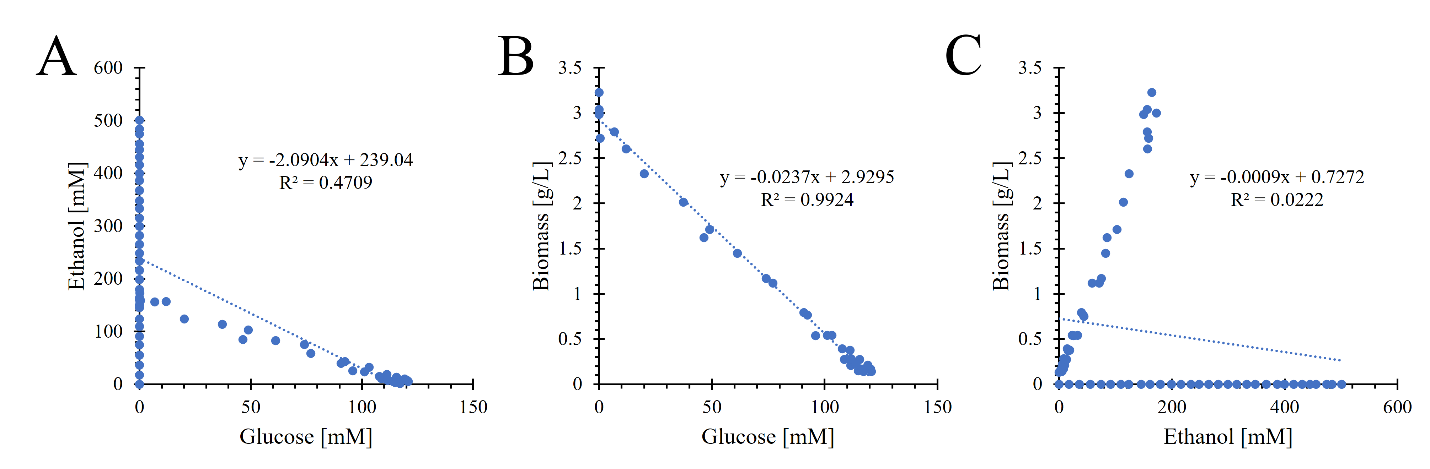


**Figure 5.5.3:** The cross-correlations in the ethanol supplemented calibration dataset represented as glucose versus ethanol (A), glucose versus biomass (B), and ethanol versus biomass (C)
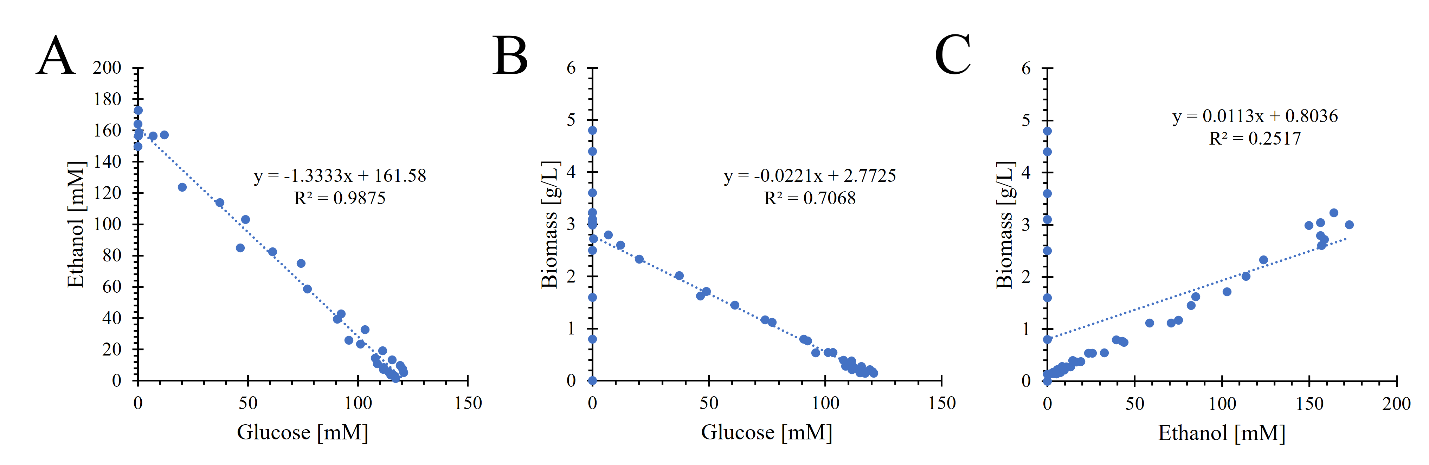
**Figure 5.5.4:** The cross-correlations in the biomass supplemented calibration dataset represented as glucose versus ethanol (A), glucose versus biomass (B), and ethanol versus biomass (C).

**5.6 Performance of supplemented models on batch validation dataset**

| **Model target** | **Calibration range** | **R^2^**  **Cal./CV/Pred. (batch)** | **RMSEC** | **RMSECV** | **RMSEP batch** | **RMSEP fed-batch** | **Latent Variables** |
| --- | --- | --- | --- | --- | --- | --- | --- |
| Glucose | 0 – 247.08 mM | 0.997/0.996/0.999 | 3.05 mM | 3.39 mM | 1.71 mM | 3.06 mM | 2 |
| Ethanol | 0 – 513.28 mM | 0.997/0.997/0.999 | 8.02 mM | 8.14 mM | 4.20 mM | 8.65 mM | 2 |
| Biomass | 0.10 – 4.81 g/L | 0.981/0.967/0.995 | 0.18 g/L | 0.24 g/L | 0.17 g/L | 0.99 g/L | 3 |

**Table 5.6.1:** Overview of statistics for the supplemented models applied to the validation batch and fed-batch datasets for glucose ethanol and biomass.

**Table 5.6.2:** Comparison table of the relative root mean square errors of calibration (rRMSEC), cross-validation (rRMSECV) and prediction (rRMSEP) of the base and supplemented (suppl.) models. The errors were normalized to the interquartile ranges of the calibration dataset (for rRMSEC and rRMSECV) or the prediction dataset (for rRMSEP).

| **Model target** | **rRMSEC base** | **rRMSEC suppl.** | **rRMSECV base** | **rRMSECV suppl.** | **rRMSEP base** | **rRMSEP suppl.** | **rRMSEP base** | **rRSMEP suppl.** |
| --- | --- | --- | --- | --- | --- | --- | --- | --- |
|  |  |  |  |  | **Applied to batch** | | **Applied to fed-batch** | |
| Glucose | 2.17 % | 3.27 % | 1.89 % | 3.63 % | 2.07 % | 2.42 % | 30.38 % | 5.25 % |
| Ethanol | 3.75 % | 3.59 % | 4.45 % | 3.65 % | 3.57 % | 3.44 % | 62.06 % | 6.17 % |
| Biomass | 4.45 % | 7.85 % | 6.99 % | 10.47 % | 5.38 % | 8.32 % | 87.76 % | 26.98 % |
